# On the Synergistic Effect of Multi-Walled Carbon Nanotubes and Graphene Nanoplatelets to Enhance the Functional Properties of SLS 3D-Printed Elastomeric Structures

**DOI:** 10.3390/polym12081841

**Published:** 2020-08-17

**Authors:** Gennaro Rollo, Alfredo Ronca, Pierfrancesco Cerruti, Xin Peng Gan, Guoxia Fei, Hesheng Xia, Gleb Gorokhov, Dzmitry Bychanok, Polina Kuzhir, Marino Lavorgna, Luigi Ambrosio

**Affiliations:** 1 Institute of Polymers, Composites and Biomaterials, National Research Council, Via Campi Flegrei, 34-80078 Pozzuoli (NA), Italy; gennaro.rollo@ipcb.cnr.it (G.R.); cerruti@ipcb.cnr.it (P.C.); luigi.ambrosio@cnr.it (L.A.); 2 Institute of Polymers, Composites and Biomaterials, National Research Council, Via Previati, 1, 23900 Lecco, Italy; alfredo.ronca@cnr.it; 3 Institute of Polymers, Composites and Biomaterials, National Research Council Viale J.F. Kennedy, 54-80125 Naples, Italy; 4 State Key Laboratory of Polymer Materials Engineering, Polymer Research Institute, Sichuan University, Chengdu 610065, China; xinpenggan@163.com (X.P.G.); feiguoxia1981@163.com (G.F.); 5 Institute for Nuclear Problems of Belarusian State University, Bobruiskaya 11, 220006 Minsk, Belarus; glebgorokhov@yandex.ru (G.G.); dzmitrybychanok@ya.ru (D.B.); polina.kuzhir@gmail.com (P.K.); 6 Physics Faculty, Vilnius University, Sauletekio 9, LT-10222 Vilnius, Lithuania; 7 Radiophysics department, Tomsk State University, Lenin Avenue 36, 634050 Tomsk, Russia; 8 Institute of Photonics, University of Eastern Finland, Yliopistokatu 7, FI-80101 Joensuu, Finland; 9 Institute of Polymers, Composites and Biomaterials, National Research Council, P. le Enrico Fermi, 1-80055 Portici (NA), Italy

**Keywords:** selective laser sintering, piezoresistivity, thermoplastic polyurethane (TPU), carbonaceous filler, EMI shielding

## Abstract

Elastomer-based porous structures realized by selective laser sintering (SLS) are emerging as a new class of attractive multifunctional materials. Herein, a thermoplastic polyurethane (TPU) powder for SLS was modified by 1 wt.% multi-walled carbon nanotube (MWCNTs) or a mixture of MWCNTs and graphene (GE) nanoparticles (70/30 *wt*/*wt*) in order to investigate on both the synergistic effect provided by the two conductive nanostructured carbonaceous fillers and the correlation between formulation, morphology, and final properties of SLS printed porous structures. In detail, porous structures with a porosity ranging from 20% to 60% were designed using Diamond (D) and Gyroid (G) unit cells. Results showed that the carbonaceous fillers improve the thermal stability of the elastomeric matrix. Furthermore, the TPU/1 wt.% MWCNTs-GE-based porous structures exhibit excellent electrical conductivity and mechanical strength. In particular, all porous structures exhibit a robust negative piezoresistive behavior, as demonstrated from the gauge factor (GF) values that reach values of about −13 at 8% strain. Furthermore, the G20 porous structures (20% of porosity) exhibit microwave absorption coefficients ranging from 0.70 to 0.91 in the 12–18 GHz region and close to 1 at THz frequencies (300 GHz–1 THz). Results show that the simultaneous presence of MWCNTs and GE brings a significant enhancement of specific functional properties of the porous structures, which are proposed as potential actuators with relevant electro-magnetic interference (EMI) shielding properties.

## 1. Introduction

Piezoresistivity is defined as the electrical resistance variation induced by an external mechanical stimulus [1]. Piezoresistive sensors are traditionally fabricated by metallic or inorganic semiconductor materials, but they are typically rigid, heavy, and brittle, and this limits their utilization in many fields [2]. To address these drawbacks, in recent years, porous polymer materials reinforced with conductive nanostructured fillers have been engineered as piezoresistive sensors. The goal is to obtain electrical conductive polymer composites by forming a three-dimensional interconnected conductive network made of conductive fillers. In this respect, metal nanoparticles [3], intrinsically conductive polymers [4] dispersed in polymer matrix [5], or carbonaceous fillers such as carbon black [6], carbon fibers [7], carbon nanotubes [8], graphite [9], and graphene [9] have been widely used. Flexibility, high porosity, ultra-low density, good energy conversion, and storage properties are the properties achieved by these nanocomposites [10]. Such conductive nanocomposites find a wide range of applications as pressure sensors [11] as well as flexible circuits [12], antistatic materials [13], and electromagnetic interference shielding devices [14].

However, constructing 3D interconnected conductive networks using conventional manufacturing such as in situ polycondensation [15], templating methods [16], melt processing [17], and solution mixing [18] is a challenging task, because the high shearing force present in these conventional processes breaks the conductive network structure. 3D printing is an innovative manufacturing technology that, based on Computer-Aided Design (CAD) data, can directly turn complex 3D models into real objects [19,20,21]. Selective laser sintering (SLS) is one of the most significant 3D printing techniques, which is applied in many fields, from biomedicine to aerospace [22]. SLS is a powder-based process in which 3D structures are obtained by the layer-by-layer sintering of thermoplastic polymer powder via a computer controlled scanning laser beam [23]. Differently from other 3D printing techniques, such as fused deposition modelling (FDM), SLS is a shear-free and free-flowing process that, in case the raw polymeric powder is coated with conductive filler, allows the formation of a segregated filler network within the polymer matrix [24].

Thermoplastic polyurethane (TPU) is a soft and flexible elastomer widely used as building material for the SLS process [25]. Recently, TPU composites reinforced with nanoscale fillers drew great attention for their enhanced mechanical, thermal, and electrical properties [26,27]. In fact, combining a TPU matrix with conductive fillers and SLS manufacturing is a simple and effective approach to prepare nanocomposite materials with optimized thermal, anticorrosive, and electromagnetic shielding properties [11,28,29,30]. Xia et al., recently developed a novel approach to construct a 3D electrically conductive segregated network in TPU/CNTs composite polymer matrix by SLS [24]. Later, they reported the simultaneous realization of conductive segregation network microstructure and minimal surface porous macrostructure by SLS 3D printing of single-walled carbon nanotubes (SWCNTs)-wrapped TPU composite powder. [31] The Schwarz (S-) structure was found to be capable of producing the best piezoresistive properties of the SWCNTs/TPU composite sensor with a gauge factor (GF) much higher than that for Gyroid and Diamond structures.

In a previous research, some of the authors have manufactured TPU/1 wt.% graphene (GE) porous systems, and demonstrated the correlation between geometrical features and electrical properties of the 3D-printed porous structures. All porous structures exhibited a robust negative piezoresistive behavior, with outstanding strain sensitivity. However, the obtained results showed that GE particles obstruct the polymer powder coalescence, thereby resulting in a porous structure that exhibits an imperfect percolative network and poor mechanical properties [27]. The analysis of the literature confirms that elastomer-based porous structures realized by SLS technology with powders modified with carbonaceous fillers have been exhaustively investigated as innovative materials for piezoresistive sensors. However, in this context, a fully understanding of the formulations–properties correlations which establish when 2D and 1D carbonaceous filler mixtures are used to modify the elastomeric-based SLS particles are still missing.

Alongside with outstanding piezoresistive behavior, the elastomer-based conductive porous structures exhibit interesting properties to be used in the field of Electromagnetic Interference Shielding. In fact, the porosity and electrical conductivity are the most important material parameters responsible for the electro-magnetic (EM) response of materials [32]. Thus, in case of high conductivity and zero porosity, like, e.g., conventional metals of valuable thickness (thicker than skin depth), electro-magnetic interference shielding efficiency (EMI SE) is high and ascribed mainly to the reflection from the topmost metal surface. For thinner than skin depth films with metallic conductivity (e.g., graphene and other carbon nm-films [33,34,35], high absorption close to 100% is possible in case of placing it to λ/4 dielectric plate or back reflector separated from the conductive film by a fine insulating slab (so-called Salisbury screen) [34,36].

In case of porous monoliths, even for a highly conductive backbone, in contrast to bulk metals, it is possible to reach resonant perfect electromagnetic absorption. For that, the void/cell size should be compatible with the wavelength [37].

However, for many applications, it is not necessary resonant, even perfect, absorption. Many applications, especially in the field of EMI shields, require high but not perfect broadband absorption. The simplest way is to use slightly conductive media of proper thickness in order to suppress the reflection due to constructive interference effects. However, such a solution demands a thick and heavy EMI shielding layer (e.g., epoxy filled with multi-walled carbon nanotube (MWCNT) above the percolation threshold must be not less than 10 mm thick and have a targeting frequency of 10 GHz [38,39]), and it again supports resonant absorption.

The advantage of Diamond (D) and Gyroid (G) lattices fabricated by SLS 3D printing from conductive polymer composites is the option to tune their geometrical features (porosity, void size) to target a particular frequency range. Moreover, the conductivity of the lattices skeleton has to be enough to ensure Joule heating (true absorption of electromagnetic waves), and to secure multiple reflection from the void/cell walls enhancing resultant absorption.

In this research, different types of carbonaceous fillers able to improve the electrical properties of the porous structures realized by SLS were investigated. In detail, two kinds of fillers were used to coat the TPU powders: multi-walled carbon nanotubes (MWCNTs) and a combination of MWCNTs and GE (70/30 *wt*/*wt*). MWCNTs are a low-cost filler, if compared to single-walled carbon nanotubes (SWCNTs), and SLS 3D printing induces their segregation, improving the conductive percolation network. Moreover, it has been demonstrated that the combined use of MWCNTs and GE allows the realization of nanocomposites with better electrical properties in terms of conductivity and gauge factor if compared to MWCNTs and GE alone. Nanocomposite TPU powders have been processed by SLS to obtain mathematically defined structures with different shapes and porosities. The effect of the filler, porosity, and geometry on the electrical and mechanical properties of the structures was evaluated, and a comparison with our previously reported research was conducted. Moreover, electromagnetic shielding characterization was performed on the porous structures that showed the best electrical properties.

## 2. Materials and Methods

### 2.1. Preparation of Nanocomposites Powder

The method of SLS-compatible composite powder preparation directly determines the dispersion of nanofillers in the polymer matrix, and this affects the structural and functional properties of the SLS-printed structures. MWCNTs (NANOCYL 7000, Nanocyl, Sambreville, Belgium) and Graphene (The Sixth Element Materials, Changzhou, China) with a MWCNTs/GE ratio of 70/30 *wt*/*wt*. were first pre-dispersed by a wet ball milling process as previously described [31]. The ball mill jars were fixed on the planetary mill and then milled continuously for 1 h at a speed of 300 rpm due to the action of iron balls in the milling jars. Anhydrous ethanol was then added to the dark dispersion and the solution was sonicated (40 W for 1 h) to obtain a stable MWCNTs-GE dispersion. The TPU powders (Mophene3D T90A, Nanjing, China) were then added to the MWCNTs-GE suspension, in such an amount to obtain a final filler content of 1 wt.%, and subjected to mechanical stirring for 2 h. The resulting mixture was filtered with a Buchner funnel under reduced pressure and dried in a vacuum oven at 70 °C for 24 h. Afterwards, the TPU/MWCNTs-GE powders were sieved to remove particles with a size over 150 µm, and silica powder was added to further improve the powder flowability. For a comparative experiment, the control sample TPU/MWCNTs composite powder (Mophene3D CT90A, Nanjing, China) was used as received.

### 2.2. Porous Structures Design and Manufacturing by SLS Technology

To design 3D porous structures, a mathematical approach was used starting from triply periodic minimal surfaces equations (TMPS). TPMS are minimal surfaces periodic in three independent directions, extending infinitely and, in the absence of self-intersections, partitioning the space into two labyrinths. The Wolfram Mathematica software was used to generate the 3D structure based on Gyroid (G) and Diamond (D) equations with different porosity. The following trigonometric equations, i.e., Equation (1) for G and Equation (2) for D structures, were used with boundary condition x, y, z = [−3π; 3π]:

(1) cos(x)·sin(y)+cos(y)·sin(z)+cos(z)·sin(x)=C

(2) sin(x)·sin(y)·sin(z)+sin(x)·cos(y)·cos(z)+cos(x)·sin(y)·cos(z)+cos(x)·cos(y)·sin(z)=C

where *C* is the offset parameter and controls the porosity of the structures. Porous structures with three different porosities (20%, 40%, and 60%) were designed to study the correlation between porosity and electrical properties. Hereinafter, Gyroid and Diamond porous structures will be labelled as Gx and Dx, respectively, where G and D represent the geometry and *x* represents the porosity in %. As an example, G20 stands for Gyroid architectures with 20% porosity. The CAD model of the specimen was generated using the Rhinoceros CAD software (Robert McNeel & Associates, WA, USA.), and exported in the STL format for uploading into the SLS machine. The SLS process was performed on a lab-scale SLS equipment (Sharebot-SnowWhite, Lecco, Italy). The optimized sintering process parameters for TPU/MWCNTs and TPU/(MWCNTs-GE) are shown in Table 1. To process the nanocomposite powder, the laser was set at 40% of the maximum energy.

The manufactured structures were allowed to cool inside the machine process chamber for approximately 1 h and then they were removed from the part bed. Excess of powder surrounding the structure and non-sintered powder from the interstices were removed by compressed air.

**Figure 1:**
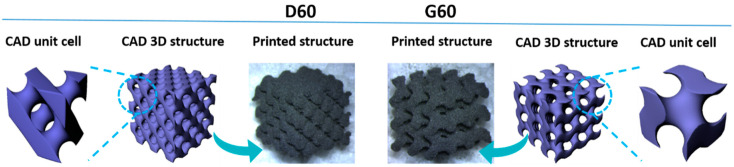
Computer-Aided Design (CAD) unit cell, CAD 3D structure, and printed for D60 and G60 systems.

Examples of D and G architectures with 60% porosity, starting from the CAD unit cell, the 3D structure and, finally, a picture of the 3D-printed samples are shown in Figure 1.

### 2.3. Electron Microscopy

Scanning electron microscopy (SEM) observations were performed by a Fei Quanta 200 SEM (Hillsboro, OR, USA) to study the morphology of the porous structures. The samples were fixed on a support and metallized with a gold-palladium alloy to ensure better conductivity and prevent the formation of electrostatic charges. Transmission electron microscopy (TEM) imaging was performed using a Tecnai G2 Spirit TWIN electron microscope (FEI, Hillsboro, OR, USA) operating at 120 kV on thin sections obtained from the bulk samples using a Leica EM UC7 ultracryomicrotome (Leica Microsystems Srl, Milano, Italy) at −100 °C, cut rate between 1 and 8 mm/s, and nominal feed 140 nm.

### 2.4. Thermal Characterization

Thermal properties of SLS-printed samples were measured by thermogravimetric analysis (TGA) using a PerkinElmer Pyris Diamond TG/DTA (Waltham, MA, USA). Approximately 8 mg of sample were first heated to 90 °C at 10 °C/min, kept in isothermal conditions for 10 min, and then heated to 800 °C at a heating rate of 5 °C/min under nitrogen atmosphere.

### 2.5. Piezoresistive Measurements

The experimental setup for the evaluation of the mechanical and piezoresistive properties of the 3D-printed porous structures consisted in a mechanical tester (Instron 5564 dynamometer, Torino, Italy) and a multimeter (Agilent 34401A 6½ Digit Multimeter, Santa Clara, CA, USA) controlled by a homemade LabVIEW program. The multimeter was set up with the 2-probe measurement method, able to continuously monitor the change in the electrical resistance of the specimen submitted to loading and unloading cycles. The top and bottom surfaces of the cubic specimens (10 × 10 × 10 mm^3^) were covered with copper conductive tape as electrode. In detail, the electrical resistance changes were evaluated by submitting the samples at room temperature (25 °C) to cyclic loading/unloading, with 8% deformation and 3 mm/min actuation rate.

### 2.6. Electromagnetic Shielding

#### 2.6.1. Low-Frequency Range

The low-frequency conductivity of G20 and G60 structures made of TPU/MWCNT and TPU/(MWCNT-GE) powders was investigated in a 100 kHz-1 MHz range in order to ensure the existence of percolation in composites. Measurements were conducted by a HP4284A LCR-meter (Zurich Instruments, Cambridge, MA, USA). Specimens of approximate ~5 × 5 × 3 mm^3^ dimensions were placed between two parallel electrodes and measured as quasi-bulk samples. The LCR-meter measures the values of capacity and loss tangent, which allows calculating the conductivity.

#### 2.6.2. Microwave Range

The electromagnetic response of structures G20 and G60 (cubic samples) containing MWCNTs and MWCNTs-GE was investigated in Ku-band (12–18 GHz) using a Micran R4M (Micran, Tomsk, Russia) vector analyzer and rectangular waveguide transmission line with cross-sectional dimensions of 16 × 8 mm^2^. Plain-parallel samples of 10.6 mm thickness were placed into the waveguide and their complex S_21_-parameters (being square root of sample transmission) were measured. The complex permittivity value was calculated from the experimental data by standard methods [40].

#### 2.6.3. THz Range

The electromagnetic response in the terahertz frequencies was measured by the time-domain spectrometer “T-Spec” by EKSPLA (Vilnius, Lithuania). The measurement procedure has been described in detail elsewhere [41]. Two millimeters thick plane-parallel slices of porous structure (7 × 5 cm^2^) were placed between emitter and detector normally to the incident EM wave. The THz detector output is proportional to the instant electrical field strength of the THz pulse during the ultrashort pumping pulse. The Fourier transform of the waveform of electrical field of THz radiation gives the frequency dependence of complex transmission and reflection coefficients. The measurements were done in both transmission and reflection modes. The absorption coefficient, A, was calculated as A = 1 – T − R, where T and R are the transmission and reflection coefficients, respectively.

## 3. Results and Discussion

### 3.1. Morphological Characterization of the Porous Structures

**Figure 2:**
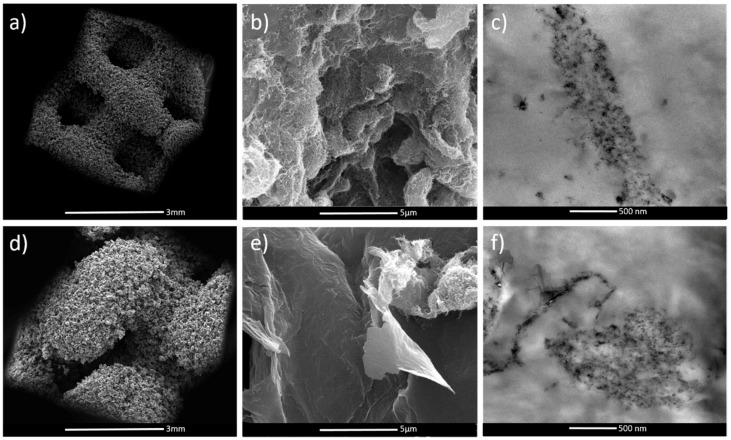
SEM of TPU/MWCNTs D60 and G60 samples at 50× magnification (**a**,**d**), SEM (**b**) and TEM (**c**) images of TPU/MWCNTs G60, and SEM (**e**) and TEM (**f**) images of TPU/(MWCNTs-GE) G60 porous structures.

SEM and TEM analysis of the porous structures (Figure 2) demonstrated that MWCNTs and GE sheets were segregated between the TPU particle boundaries, forming a percolated conductive network. Figure 2a,d reports the SEM images of TPU/MWCNTs D60 and G60 porous structures at low magnification. It is possible to see the differences in pore structures and thickness of internal trabeculae of D and G geometries as obtained by the SLS process. In particular, it is quite evident that the trabeculae of the G geometry are bigger than the trabeculae of the D geometry (about 30% bigger, as also discussed later in the paper). Indeed, the sintering of the TPU nanocomposite particles results in the formation of a continuous filler path spanning within the polymer matrix [24]. More specifically, SEM images (Figure 2b,e) clearly demonstrate that the surface of the TPU particles are covered with MWCNTs and GE filler particles. TEM pictures (Figure 2c,f) illustrate the formation of the percolated network due to the filler confinement between the sintered TPU particles, with a thread thickness ranging from 200 to 500 nm. It is worth noting that in the sample TPU/(MWCNTs-GE) G40, it is possible to observe that both fillers (MWCNTs and GE) were trapped between the polymer particles, forming a stable percolative network with a low filler concentration (i.e., 1 wt.%).

### 3.2. Thermal Properties

**Figure 3:**
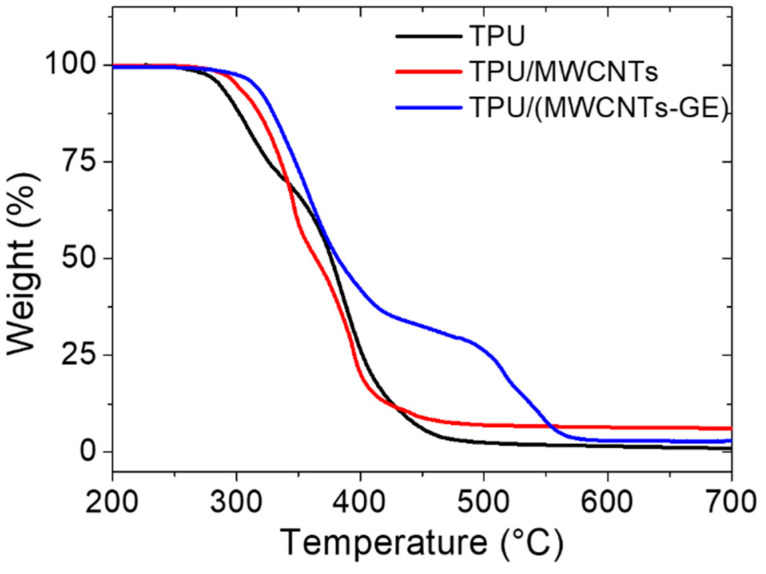
Thermogravimetric analysis (TGA) of TPU (black), TPU/MWCNTs (red), and TPU/(MWCNTs-GE) (blue).

Thermal characterization of TPU-based samples was performed by TGA analysis. The thermogravimetric curves of TPU-based samples are compared in Figure 3. TPU degradation occurs in two steps as already described in our previous research [27]. Briefly, the first degradation that starts at about 280 °C is attributed to the cleavage of urethane bonds of TPU [42] and shows a maximum rate at 309 °C, accounting for about 30% mass loss. The second weight loss, with a maximum rate at 387 °C, is related to the decomposition of soft segments of TPU leading to a residual char value of 1.2%. The presence of the MWCNTs filler affects both the degradation onset, which occurred at about 300 °C, and the degradation rate maximum, which shifted to 340 °C. For the mixed system TPU/(MWCNTs GE), a dramatically different degradation curve was recorded with respect to the pristine TPU.

The first degradation step started at around 310 °C, followed by a second degradation step at 488 °C. Therefore, the addition of fillers brings about an improvement of thermal stability of TPU, in particular, there is a synergistic effect of the CNTs and GE in the system with mixed fillers [43].

### 3.3. Mechanical and Piezoresistive Characterization

**Figure 4:**
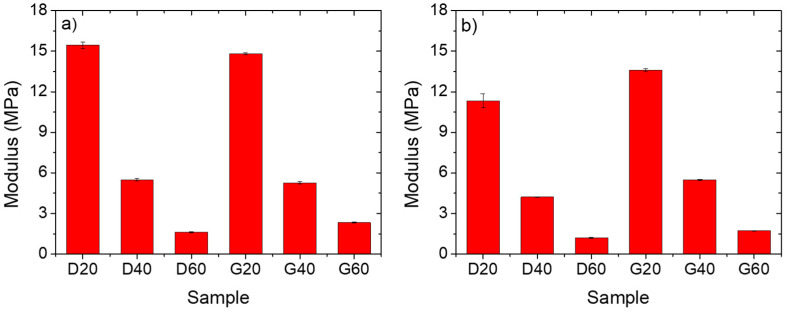
Compression elastic modulus of the selective laser sintering (SLS)-fabricated TPU/MWCNTs (**a**), and TPU/(MWCNTs-GE) (**b**) porous structures.

Compression tests were performed to study the effects of porosity and geometry on the mechanical behavior of the 3D porous structures. The samples were tested at small strain values (<10%) in order to consider the behavior in the linear elastic region. The values of elastic modulus are reported in Figure 4.

The average thickness of the trabeculae for TPU/MWCNTs and TPU/(MWCNTs-GE) systems with comparable geometry is similar, and this explains the comparable results above all for the systems with G unit cell geometry. As expected, raising the percentage of porosity causes the elastic modulus to strongly decrease, going, for the systems containing MWCNTs, from 15 MPa of the D20 to the 1.5 MPa of the D60. This is easily understandable by thinking that structures with higher porosity are characterized by thinner internal trabeculae, and this in turn strongly affects the mechanical response [31]. Moreover, it can be assessed that the type of filler seems to influence the mechanical response of the samples. In fact, the MWCNTs-based systems show better mechanical properties if compared to the MWCNTs/GE-based ones. This can be ascribed to the presence of GE nanosheets on the TPU particle surface that prevent the coalescence of the particle during the sintering process, thereby reducing the mechanical properties of the porous structure. This was confirmed also by comparing the mechanical properties of the proposed systems with the TPU/GE systems that we reported in a previous paper, where for the D60 system the elastic modulus value was 1.4 MPa [27].

**Figure 5:**
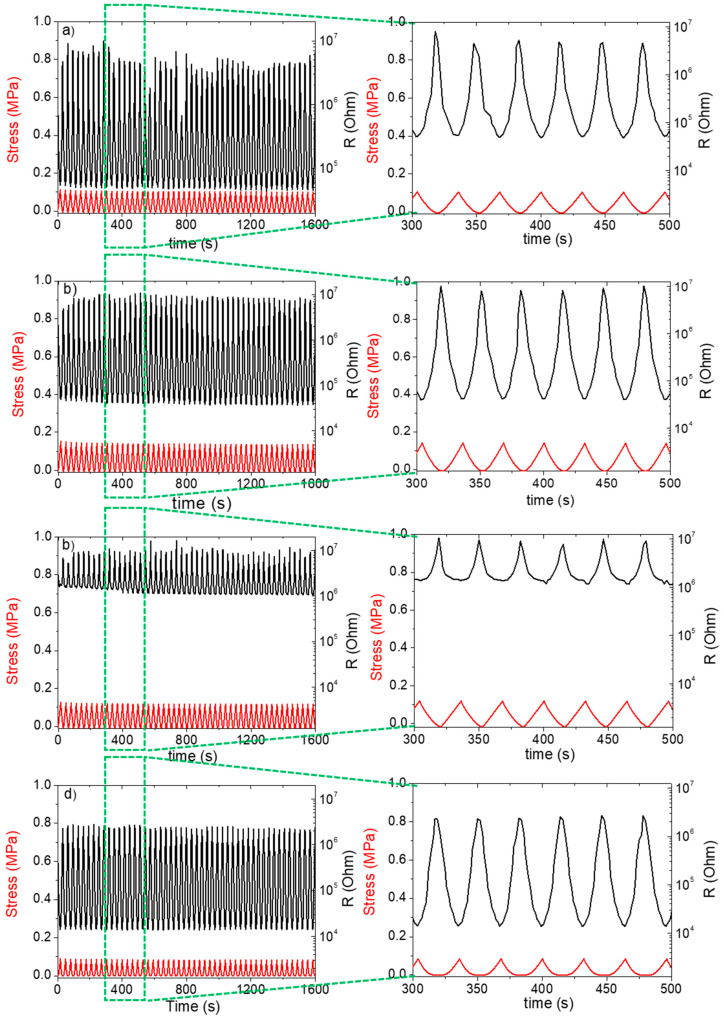
Piezoresistive behavior in terms of variation of compression stress and electrical resistance for the systems of (**a**) TPU/MWCNTs with D geometry, (**b**) TPU/MWCNTs, (**c**) TPU/GE, and (**d**) TPU/(MWCNTs-GE) with G geometry. All systems have 60% porosity and are submitted to compression loading/unloading cycles of up to 8% strain.

The piezoresistive behavior of the 3D-printed porous structures was studied by submitting the samples to strain-controlled compression cycles with a maximum strain of 8%. Alongside with the mechanical response, the electrical resistance (R) of the structures was measured as a function of the compressive strain. The samples were submitted to 50 compressive cycles and the results of the TPU/MWCNTs systems with 60% of porosity are reported in Figure 5. By comparing the data shown in Figure 5a,b, it is possible to assess the effect of the geometry for the systems with the same formulation. The R values are similar, but the variation of the electrical resistance, ΔR, as consequence of the mechanical compression, is larger for the sample with G geometry. In the cyclic compression process, the mechanical response of the sample was very stable, whereas the electrical response presented some slight instability (which could also be ascribed to the electrical contact between the sample and the electrode) all over the experiment. However, the results confirmed the signal reversibility, with a direct correlation between the strain and the electrical resistance, that decreased by increasing the compression strain.

A direct comparison between the effects of different fillers on the piezoresistive behavior of printed samples with the same geometry and porosity is useful to give evidence of a possible synergistic effect. For sake of comparison, in this context, it has been considered important to present also the results related to the systems realized with GE nanoplatelets that were the object of a previous paper [27]. Electrical resistance of the TPU/GE system, after compression at 8% strain (Figure 5c), was higher than that of the MWCNTs system (Figure 5b). This behavior is in agreement with the literature data and the lower conductivity of GE-based composites as compared with the MWCNTs composites [44,45].

**Figure 6:**
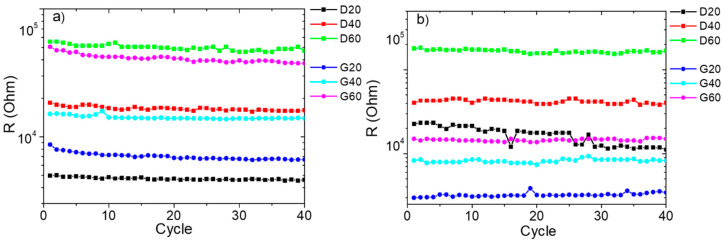
Resistance values at 8% strain for TPU/MWCNTs (**a**) and TPU/(MWCNTs-GE) (**b**) porous structures during the compression loading/unloading cycles.

Moreover, it was possible to observe a synergistic effect of the fillers in the TPU/(MWCNTs-GE) system (Figure 5d). In fact, the resistance values for the TPU/(MWCNTs-GE) system at 0% and 8% strain were lower than those of the TPU/MWCNTs composite. This can be ascribed to the formation of more conductive pathways [46]. Furthermore, as it was expected, the resistance at 8% strain depends on sample porosity and geometry (see Figure 6) resulting larger for the systems with higher porosity (more details are provided later). Finally, the electrical resistance does not change during loading/unloading cycles as consequence of the satisfying robustness and stability of the porous structures.

**Figure 7:**
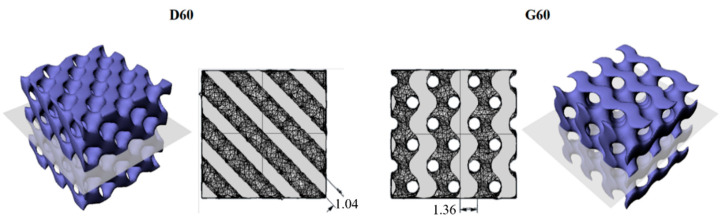
Cross-section areas and thickness of trabeculae for D60 and G60 structures.

It is worth noting that for the TPU/(MWCNTs-GE) composite, the systems with Gyroid structures showed significantly lower electrical resistance when subjected to compressive strain. This can be ascribed to the different internal structures of D60 and G60 samples. As shown in Figure 7, the cross-section area of G60 present thicker trabeculae if compared with D60 geometries (as also confirmed by SEM images reported in Figure 2. In fact, the average thickness of the G60 structure trabeculae is 1.360 ± 0.001 mm, which is 30% higher compared with the thickness of the D60 structure (equal to 1.040 ± 0.001 mm). This means that in the G60 structure, during compression loadings, the fillers create more effective percolating networks by forming more MWCNTs and GE nanoplatelets contacts.

To compare our results with those found in literature, it is worth noting that the average ΔR/R_0_ value measured for our systems is equal to 99.4% with a compression strain of 8%. Kang et al., measured a ΔR/R_0_ equal to 0.8% for a pressure of 5 MPa for systems consisting of single-wall-carbon nanotube/Polyimide, which is one order of magnitude higher compared with the compression stress applied in this research (i.e., about 0.2 MPa as shown in Figure 5 and Figure 6) [47]. Similarly, a value of ΔR/R_0_ ≈ 15%, that is still lower as compared to the one shown by the systems investigated in this paper, at similar deformation (ε ≈ 8–10%) was reported by Ku-Herrera et al. for poly (vinyl ester) filled with 0.3 wt.% of multi-wall carbon nanotubes (PVE-MWCNT) [48]. Bao et al., found that PDMS-SWCNT materials could have a resolution of minimum detectable stress in compression of 50 KPa and report a ΔR/R_0_ value of 8% for a strain of 50% [49]. Similar results are reported for a system of poly(isoprene)-reduced graphene oxide PI-RGO [50]. The comparisons with the above-mentioned systems allows us to conclude that the approach exploited in this paper, which combines nanocomposite powder and SLS printing technology, reveals that nanocomposite sensors are extremely sensitive to deformation, with a reproducible and stable piezoelectric behavior.

In order to gain a deeper understanding of the effect of geometry, SLS printing technology, printing resolution, and formulations on the electrical resistance of the considered systems, the resistivity of materials was calculated by taking into account the porosity and the measured resistance values at 0% strain. The calculations were performed by relating the measured electrical resistance only to the bulk materials, and considering the porosity as the empty fraction volume of the total volume of samples submitted to the electrical characterization, following the model developed by Montes et al. [51]. They analyzed the problem of the electrical conduction in powdered systems and proposed an equation for computing the effective electrical resistivity of sintered aggregates (Equation (3)).

(3) $$\rho =R\cdot \left(\frac{S}{l}\right)\cdot \sqrt{{\left(1-\theta \right)}^{3}}$$

where *ρ* is the resistivity of the porous system, *R* is the calculated resistance of the porous system, *S* is the contact surface between electrode and sample (in this case correspond with the surface of the sample), *l* is the distance between the electrodes (in this case correspond with the side of the sample), and *θ* is the porosity values.

**Figure 8:**
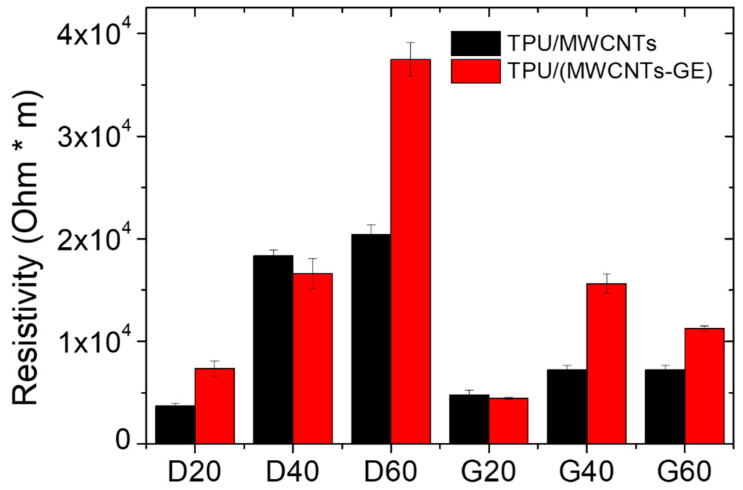
Electrical resistivity calculated by accounting for both the porosity of the systems and the electrical resistance (at 0% of strain).

Figure 8 shows the resistivity values for the TPU/MWCNTs and TPU/(MWCNTs-GE) systems, calculated accordingly with Equation (3).

The values of resistivity, which should be constant being the resistivity an intrinsic property of the materials, confirm that both the SLS printing process and geometry have a significant effect on the electrical resistance of the proposed systems. In fact, for the G samples, the resistivity is somewhat constant and does not depend on the porosity, in the range of approximations, due to the adoption of a very simple model to account for the porosity of samples. On the other side, for the systems with D geometry, the resistivity increases significantly with the porosity, confirming that the SLS printing process affects the formation of the conductive network that becomes worse and worse by increasing the porosity. This can be ascribed to the dimension of the trabeculae, which result smaller for the geometry D and give rise to a percolation network with less effective contact points between the MWCNTs and GE nanoparticles. Thus, the comparison of the electrical resistance values of systems with G geometry is robust and the variation can be related to the porosity, whereas for the D systems, it is worth considering that the geometry and SLS printing resolution affect the electrical resistance along with the porosity.

**Figure 9:**
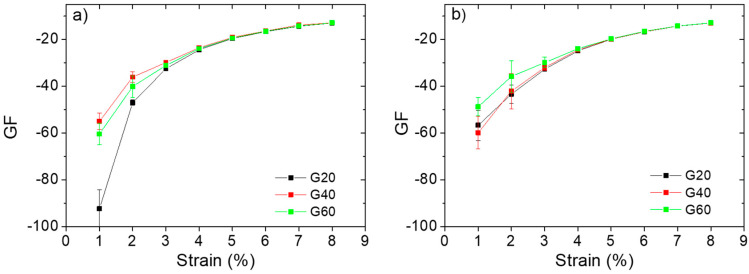
Variation of the gauge factor as a function of compression strain for TPU/MWCNTs (**a**) and TPU/(MWCNTs-GE) structures (**b**) with Gyroid unit cells.

The compression sensitivity of several porous structures was evaluated by measuring the gauge factor (GF), defined as the ratio between the relative change of the electrical resistance of the composites and their initial resistance, divided by the applied strain. All samples displayed high absolute values of GF, and, in the range of errors, no significant differences can be noticed between TPU/MWCNTs and TPU/(MWCNTs-GE) nanocomposite structures. It has to be pointed out that GF values are higher for a strain below 8%, confirming the valuable feature of the composite structures to detect small deformations. In particular, the G20 TPU/MWCNT structure showed an almost double GF value at 1% deformation, suggesting its possible use in very sensitive strain sensing devices. For all samples, GF tended to a plateau as the maximum strain value was approached, as shown in Figure 9. The presence of the plateau at high compression deformation is to be ascribed to the densification of the conductive pathways, which do not further change with the compression.

### 3.4. EM Characterization

**Figure 10:**
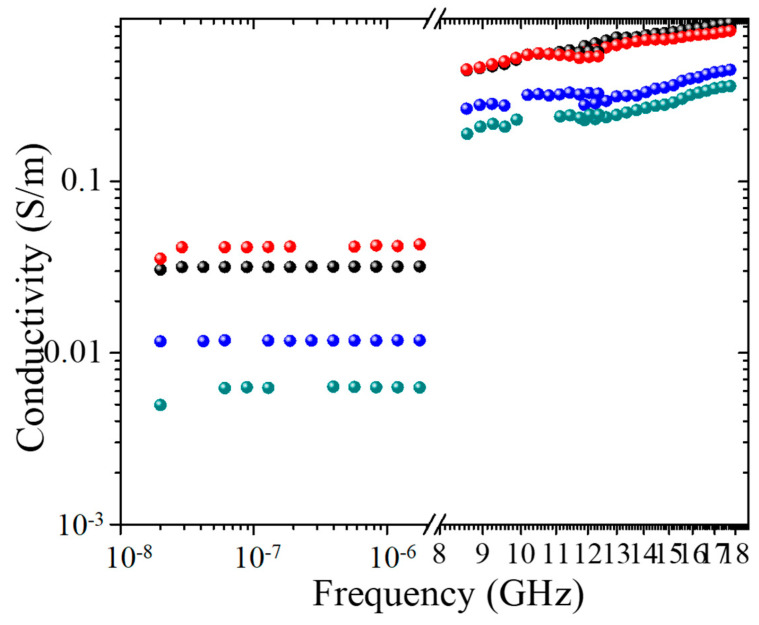
Broadband conductivity of porous structures: DC-like (0.02 kHz–1 kHz) and dielectric (12–18 GHz) conductivity behavior for TPU/MWCNTs G20 (black), TPU/MWCNTs G60 (blue), TPU/(MWCNTs-GE) G20 (red), and TPU/(MWCNTs-GE) G60 (green).

The broadband conductivity of investigated samples is shown in Figure 10. All percolated materials possess a similar frequency dependence of conductivity, consisting of two regions: the DC-like frequency-independent region is observable at lower frequencies, while at higher frequencies, the *σ~ω ^α^* dependence exists. For both TPU/MWCNT and TPU/(MWCNT-GE) structures, the presence of the DC-like conductivity at low frequencies is an evidence of percolation.

It is also possible to mention that in the DC-like range, for the systems with high porosity, the conductivity of TPU/MWCNT samples is higher than that of the systems based on TPU/MWCNT-GE, whereas only for the systems with low porosity, it seems that the GE nanoplatelets have a positive effect allowing an increment of the conductivity. The discrepancy found by comparing these results with those reported in Figure 5, may be ascribed to the fact that for broadband conductivity measurements were used with smaller samples (~5 × 5 × 3 mm^3^) as compared to those used for piezoresistive characterization. That, above all for samples with high porosity (60%), may affect the reproducibility of the results.

**Figure 11:**
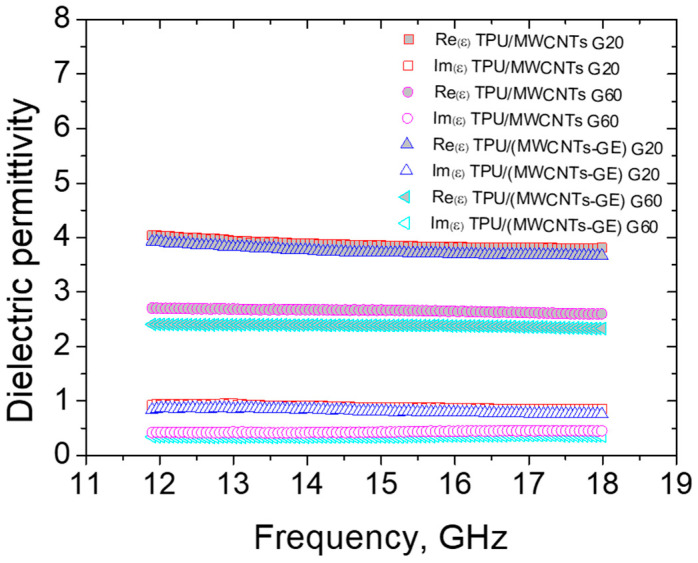
The dielectric permittivity of TPU/(MWCNTs-GE) G20 and G60 samples.

The electromagnetic shielding performance of the SLS-printed porous structures was evaluated for the G20 and G60 made of TPU/MWCNTs and TPU/(MWCNTs-GE) systems. The frequency dependence of real and imaginary parts of dielectric permittivity is presented in Figure 11.

The dielectric permittivity of all samples remains almost constant within 12–18 GHz. A minor decrease of both components of dielectric permittivity (ε) is observed for the denser sample G20. The higher values of permittivity of sample G20 vs. G60 (dense vs. lighter) are in good correspondence with the effective medium Maxwell Garnett model for composite containing conductive particles [52].

**Figure 12:**
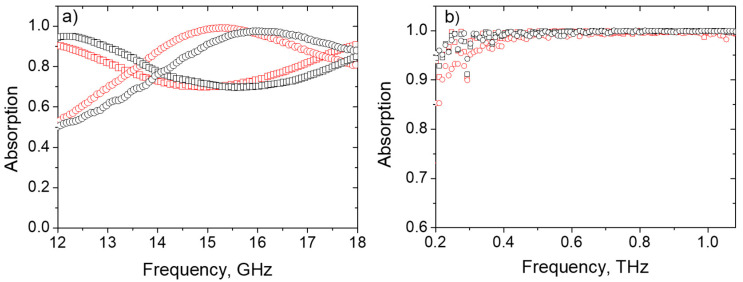
(**a**) Absorption coefficient of 10.6 mm thick samples with back reflector in the waveguide. (**b**) Absorption coefficient of 2 mm thick samples in the free space for TPU/MWCNTs G20 (red square), TPU/MWCNTs G60 (red ring), TPU/(MWCNTs-GE) G20 (black square), and TPU/(MWCNTs-GE) G60 (black ring).

The observed values of dielectric permittivity are suitable for the effective absorption of electromagnetic waves in both free space and the waveguide combined with the mirror (back reflector) [53]. According to Figure 12a, the microwave absorption coefficient within the Ku-band (12–18 GHz) is in the range of 0.51–0.99 and 0.70–0.91 for 10.6 mm thick samples of G60 and G20 series, respectively, with the peak absorption being close to 100% at 15–16 GHz.

The absorption coefficients of all investigated samples were calculated in the frequency range 0.2–1 THz (see Figure 12b) from the data collected for transmission and reflection by THz time-domain spectroscopy.

All samples (G20 and G60 series) are very lossy in the THz range and demonstrate outstanding absorption ability: the absorption coefficient of 2 mm thick samples is close to 100% starting from 300 GHz.

All investigated samples show not only extensive EMI SE, but also very high efficiency as EM waves absorbers in broad frequency range spanning from tens GHz to 1 THz. The reason is that SLS-printed samples made of TPU/MWCNTs and TPU/(MWCNTs-GE) comprise three levels of “porosity”. The inherent pores with the size coming from the lattice parameters is of 0.1 mm order (corresponding to the THz wavelength). Multiple reflection from the sides of these pores followed by Ohmic losses of the structure skeleton are the reasons of high absorption ability of G20–G60 in the THz range.

The second level is the porosity of the systems, easily visible in the SEM images (Figure 2). It corresponds to 50–100 nm pores originated by defects in the sintering of wrapped TPU particles, and because of their small size are “invisible” for both investigated microwave and THz radiation, just making the overall structure slightly lighter.

To summarize, due to nested “Russian doll” porosity structure, it is possible to approach very high absorption in different frequency ranges with one sample. Moreover, this is the way of tailoring EMI SE (absorption) addressing many frequency slots, i.e., just changing the pore size by 3D printing, porosity of the structure skeleton, and geometrical features/carbonaceous filler properties of the segregated network.

## 4. Conclusions

Porous conductive 3D structures were successfully fabricated by SLS using TPU powder wrapped with MWCNTs and a mixture of MWCNTs and GE nanofillers. The samples had a porosity ranging from 20% to 60% and were realized with the Gyroid and Diamond unit cell. Mechanical, electrical, and electromagnetic properties were investigated and correlated with porosity and internal architecture of printed samples.

SEM and TEM characterization clearly indicated that SLS manufacturing is suitable to create a high pore interconnectivity. Moreover, upon processing, the nanofillers remain segregated between the particle boundaries, forming a conductive network that facilitates the electrical percolation. The presence of GE improves the thermal stability of TPU. Compression tests and electrical conductivity measurements revealed a correlation between geometrical features and elastic modulus as well as a gauge factor. In particular, G structures showed higher elastic modulus in comparison to the D architectures.

Moreover, MWCNTs-based structures displayed satisfying electrical properties, and a synergistic conductivity enhancement was observed for the TPU/(MWCNTs-GE)-based G architectures. This was ascribed to the structure of samples with G geometry, which present bigger trabeculae and thus a better percolating network as compared to the systems with D geometry. All structures showed robust piezoresistivity, with a gauge factor value of −13 at 8% strain for all systems, which remarkably varied from −70 to −20 over strain extents ranging from 1% to 5%, which is the strain range in which the composite can be used as a sensor.

Finally, a high level of EMI SE, caused by absorption of electromagnetic waves in Ku-band (12–18 GHz), was observed for G-type samples having different porosity. The waves from 300 GHz to 1 THz could not pass through 2 mm thick G20 (60) lattice due to perfect absorption. EM response peculiarities have been associated with the multi-level porosity of the samples (starting from their cellular SLS-printed structure and due to the MWCNT/GE segregated percolative network).

Highlights: The results demonstrate that mixing MWCNTs and GE minimizes the coalescence issue, which was observed in literature for GE systems.

The right balance between mechanical and functional properties of the printed structures make these systems suitable as stable piezoresistive sensors.

The systems have relevant EMI shielding properties.

## Figures and Tables

**Table 1:** Sintering parameters adopted to process the nanocomposite powders (thermoplastic polyurethane (TPU)/multi-walled carbon nanotubes (MWCNTs) and TPU/(MWCNTs-graphene (GE))).

Process Parameters	Value
Laser power (W)	14
Laser scan spacing (μm)	200
Laser scan speed (pps)	40,000
Part bed temperature (°C)	85
Outline laser power (W)	5.6
Layer thickness (μm)	100
